# *In situ* characterization of mixed-wettability in a reservoir rock at subsurface conditions

**DOI:** 10.1038/s41598-017-10992-w

**Published:** 2017-09-07

**Authors:** Amer M. Alhammadi, Ahmed AlRatrout, Kamaljit Singh, Branko Bijeljic, Martin J. Blunt

**Affiliations:** Department of Earth Science and Engineering, Imperial College London, London, SW7 2AZ United Kingdom

## Abstract

We used X-ray micro-tomography to image the *in situ* wettability, the distribution of contact angles, at the pore scale in calcite cores from a producing hydrocarbon reservoir at subsurface conditions. The contact angle was measured at hundreds of thousands of points for three samples after twenty pore volumes of brine flooding.We found a wide range of contact angles with values both above and below 90°. The hypothesized cause of wettability alteration by an adsorbed organic layer on surfaces contacted by crude oil after primary drainage was observed with Scanning Electron Microscopy (SEM) and identified using Energy Dispersive X-ray (EDX) analysis. However, not all oil-filled pores were altered towards oil-wet conditions, which suggests that water in surface roughness, or in adjacent micro-porosity, can protect the surface from a strong wettability alteration. The lowest oil recovery was observed for the most oil-wet sample, where the oil remained connected in thin sheet-like layers in the narrower regions of the pore space. The highest recovery was seen for the sample with an average contact angle close to 90°, with an intermediate recovery in a more water-wet system, where the oil was trapped in ganglia in the larger regions of the pore space.

## Introduction

Wettability, or the contact angle between two fluids at a solid surface, determines, for instance, the efficiency of fuel cells, the security of geological carbon dioxide storage, hydrocarbon recovery, and gas exchange in leaves^1^. In the context of oil recovery, it controls the pore-scale arrangement of fluids in the rock which in turn affects macroscopic multiphase flow properties, such as relative permeability and capillary pressure^2–6^. Contact angle is defined through the Young equation,

1 $${\sigma }_{s1}={\sigma }_{s2}+{\sigma }_{12}\,\cos \,\theta $$

where σ is the interfacial tension, the subscript *s* labels solid, and 1 and 2 label the two fluid phases: the contact angle *θ* is conventionally measured through the denser phase (phase 2, or water in this paper)^2, 7^. However, traditionally, wettability is only inferred indirectly through core-scale measurements of capillary pressure and recovery to calculate average wettability indices^8, 9^. Contact angle can be measured directly, but *ex situ* on flat mineral surfaces outside the rock^4, 10–12^. Neither core-scale nor *ex situ* measurements account for pore-scale rock properties such as surface roughness, pore geometry, and rock chemical composition. Furthermore, it is important to understand how the contact angle is affected by temperature and pressure. The contact angles themselves are vital inputs into pore-scale models that predict averaged flow properties of the rock^13–15^. Furthermore, if the link between contact angle, fluid distribution, multiphase flow and recovery can be made, it should be possible to manipulate wettability in the reservoir, through the judicious choice of injection brine, for instance, to enhance oil recovery^16^. The advent of three-dimensional X-ray non-destructive imaging and image analysis at the pore-scale^17^ has enabled the identification of dynamic displacement effects and flow patterns^18–22^. However, this work has been limited to uniformly-wet systems: while the effect of wettability on flow patterns has been studied in micro-model experiments^23^, and spherical beads^24^, this has not been linked to the likely distribution of contact angle in a reservoir rock. The pore-scale interfacial curvature has been determined from pore scale imaging and compared to measurements of capillary pressure^25^. Andrew *et al*.^26^ developed a method to measure contact angle *in situ* at subsurface conditions. They manually measured contact angles for a CO_2_/brine system which was found to be weakly water-wet with angles of 45° ± 6°. Since this work, the same approach has been used to study wettability in a quarry calcite, where the solid surfaces were aged with organic acid^27^, and a CO_2_/brine system in a core that had been aged in crude oil^28^. Other authors^29, 30^ have measured receding and advancing contact angle distributions during steady-state flow in Berea sandstone confirming water-wet conditions. The contact angle distribution in bead pack and quarry calcite has been quantified using an automated algorithm^31–33^. Contact angle has also been measured manually at the nano-scale using a Cryo-BIB-SEM technique^34, 35^. However, the method is destructive and only a few measurements can be made on a sample that has been frozen.

It has been hypothesized that the typical wettability state in a reservoir is mixed-wet where regions of the pore space are water-wet while others, through direct contact with surface active components of the crude oil, become oil-wet^36–38^. However, no direct evidence for this has been available hitherto, through *in situ* measurements of contact angle for a reservoir rock/crude oil/brine system at the elevated temperatures and pressures encountered in deep subsurface formations.

In this work, mixed-wettability – defined as local effective contact angles measured *in situ* that are both less than and greater than 90° – is observed in a carbonate reservoir rock sample, extracted from a large producing hydrocarbon reservoir located in the Middle East, under subsurface conditions. We present the methods used to measure *in situ* contact angle, the results obtained, as well as associated analysis of contact angles obtained *ex situ* and compositional analysis of oil-contacted surfaces, before discussing the implications of our results.

## Results and Discussion

Our main findings of the *in situ* wettability characterization, its impact on oil recovery and the primary cause of wettability alteration are discussed in the following sections.

### *In situ* wettability characterization in weakly water-wet and mixed-wet rocks

The wide range of contact angles observed in the reservoir rock samples were characterized by applying the automated algorithm developed by ref. 32 on segmented three-dimensional images acquired by an X-ray micro-tomography scanner with a voxel size of 2 µm. This resolution is sufficient to capture the remaining oil ganglia at the micron scale and measure the effective contact angle that is essential for pore-scale models to predict average flow properties, such as relative permeability and capillary pressure. The automated algorithm eliminates the subjectivity of the manual method and enables the rapid estimation of hundreds of thousands of contact angle points.

**Figure 1:**
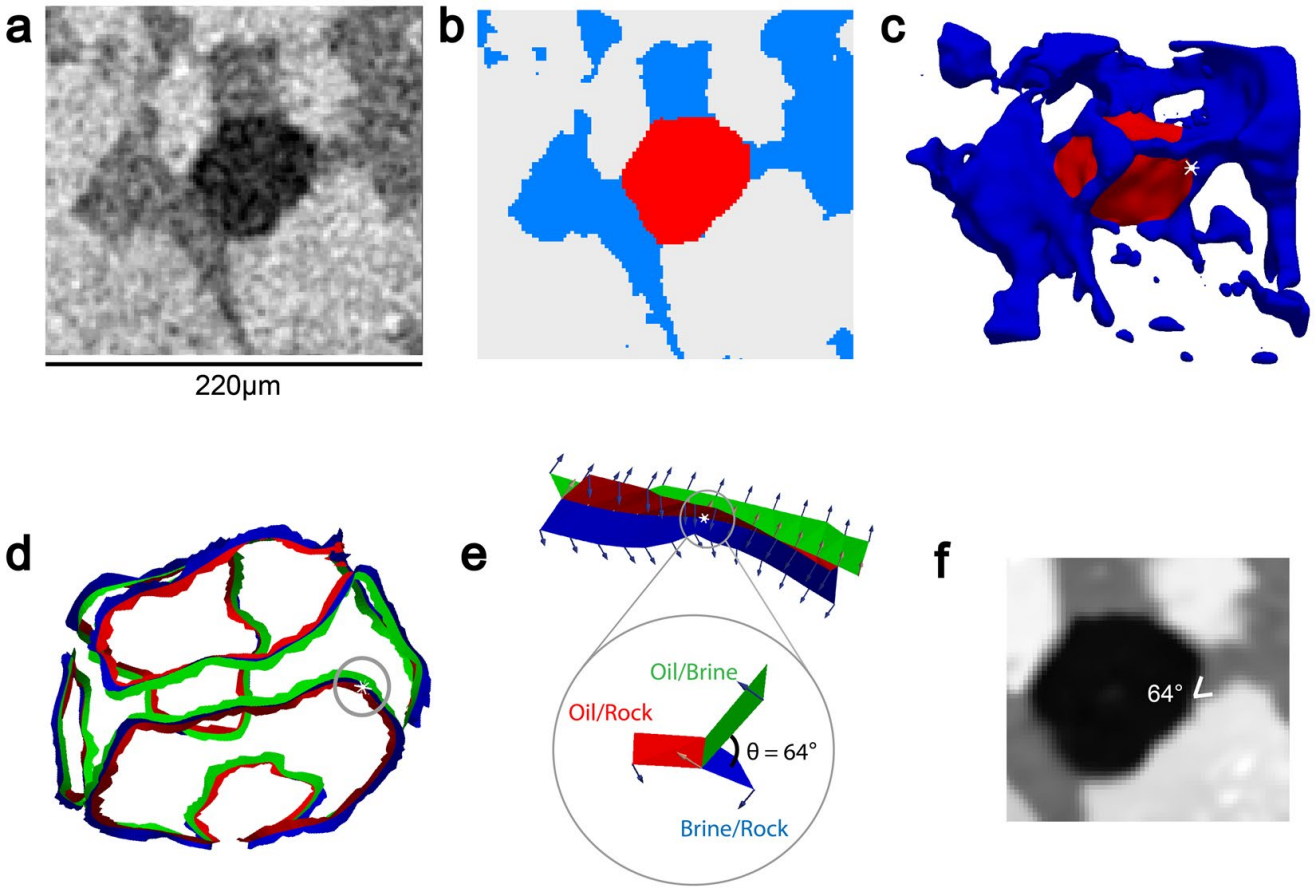
Example of an automatic contact angle measurement. (**a**) Raw image of a weakly water-wet sub-volume containing a single trapped oil ganglion with oil, brine and rock in black, dark grey and light grey respectively. (**b**) Segmented image with oil, brine and rock in red, blue and white respectively. (**c**) Three-dimensional image of generated smoothed surfaces by the automated algorithm, showing the trapped oil ganglion in red and surrounding brine in blue with a selected measured point at the white dot. (**d**) Three-phase contact line with the same point highlighted by the grey circle. (**e**) Magnified view of the three-phase contact line highlighted in (**d**) showing vectors to the interfaces, and a magnified view of the measured contact angle example of 64°. (**f**) The same measurement is superimposed on the filtered image at the same location.

An example of contact angle measurement using the automated algorithm in a sub-volume extracted from the weakly water-wet case is shown in Fig. 1. The raw image is segmented into three phases as oil, brine and rock in red, blue and white respectively. The fluid/fluid and fluid/solid surfaces were meshed and smoothed to remove voxelization artefacts. The contact angle is measured by vector dot products along the three-phase contact line. For more detail about the method please refer to the Materials and Methods section.

**Figure 2:**
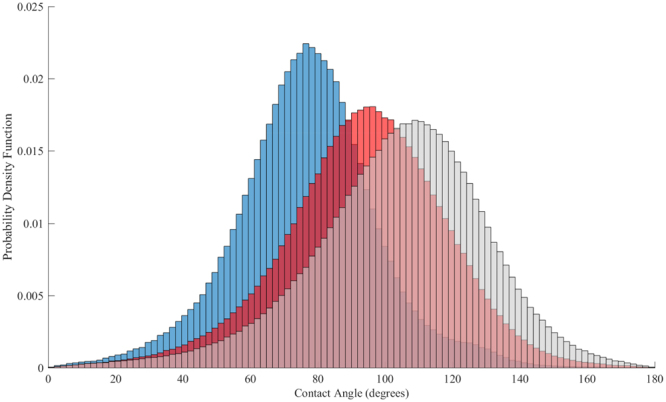
Normalized histograms of measured *in situ* contact angle distributions for sample 1 (462,000 points), sample 2 (1.41 million points) and sample 3 (769,000 points) in blue, grey and red respectively.

We applied the automatic method to all three images with a size of 976 × 1014 × 601 voxels (total of 595 million voxels) and obtained the distributions shown in Fig. 2: for sample 1 we had 462,000 measurements with a mean and standard deviation of 77° ± 21° indicating a weakly water-wet condition with few oil-wet surfaces. For sample 2, 1.41 million measurements were made with angles 104° ± 26° showing a mixed-wet system with more oil-wet surfaces; and 769,000 values were obtained for sample 3 with angles 94° ± 24° illustrating a mixed-wet system with contact angles ranging from water-wet to oil-wet.

**Figure 3:**
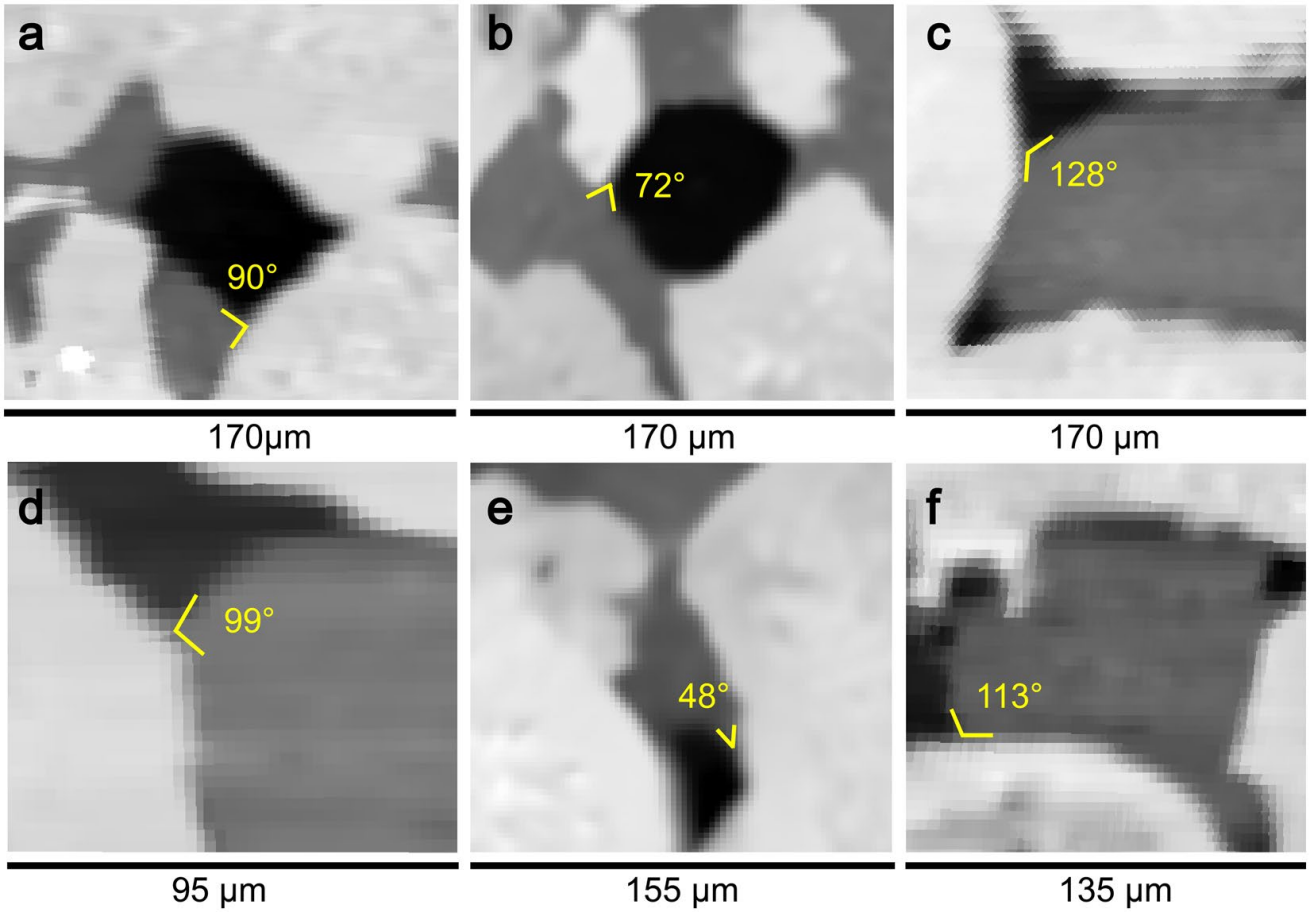
Two-dimensional cross-sections of three-dimensional images showing the range of measured contact angle. (**a**,**b**) are from sample 1, (**c**,**d**) from sample 2 and (**e**,**f**) from sample 3. Oil, brine and rock are shown in black, dark grey and light grey respectively.

Pore-scale images showing the range of contact angles are shown in Fig. 3a and b are from sample 1 showing trapped oil ganglia in the center of pores as a non-wetting phase. Fig. 3c and d are from sample 2 and Fig. 3e and f from sample 3; they show the injected brine in the center of the pore as a non-wetting phase leaving oil in pore corners and crevices. In samples 2 and 3, wettability had been altered to oil-wet in most of the pores filled with crude oil after primary drainage while un-invaded small pores, pore corners and crevices filled with brine remained water-wet. Furthermore, some regions filled with crude oil remained water-wet or had contact angles only slightly above 90° which suggests that water coated parts of the rock surfaces and might be retained within the rock surface roughness and adjacent micro-pores. Where there is no direct contact of the oil with the surface, it is unlikely to alter the rock wettability^39^.

**Figure 4:**
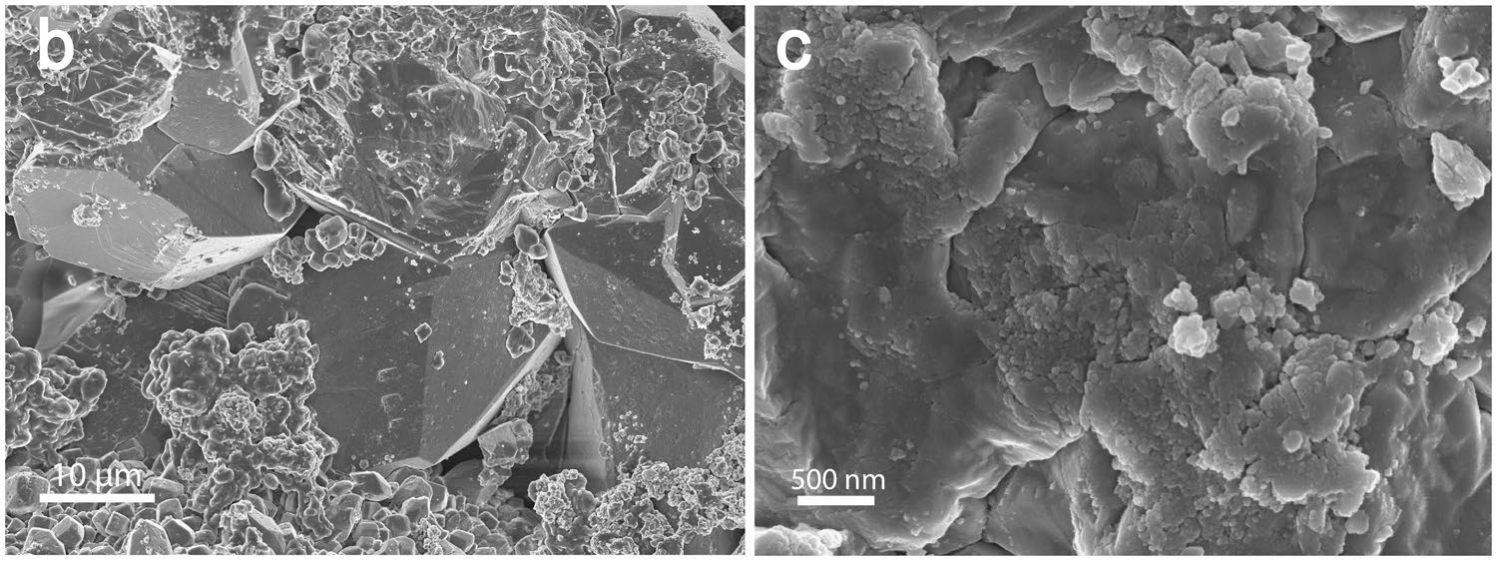
SEM images of the rock surface roughness in sample 3 at two locations (**b**) and (**c**) at different scales showing very rough surfaces with heterogeneous morphology.

SEM was used to image the rock surface roughness at two locations (B) and (C) on grain surfaces of sample 3, Fig. S4. SEM images at these two locations are shown in Fig. 4. The apparent surface roughness of the reservoir rock at the micro-scale is shown in Fig. 4b, while roughness even at the nano-scale is shown in Fig. 4c; brine can reside in this roughness preventing oil contact over much of the surface resulting in a range of contact angles even in a rock of uniform mineralogy. Furthermore, variations in contact angle could be due to hysteresis between water advancing and receding values, which is affected by the rock surface roughness^40^. The measured distribution of contact angle shown in Fig. 2 depends on how the images are segmented, since the automated algorithm^32^ can only be applied on segmented images. For more details about the impact of segmentation and filtering procedure on the estimated contact angle distribution, please refer to the Supplementary Information.

Contact angle was also measured on flat calcite mineral surfaces aged in the absence of brine. Untreated surfaces had a mean *ex situ* contact angle value of 61°. After aging *ex situ* contact angle values of 76°, 141° and 130° were measured for samples 1, 2 and 3 respectively; see the Supplementary Information. These values are at the upper end of the distributions shown in Fig. 2. This is to be expected, since we have allowed direct contact of the crude oil with the surface in the absence of brine. This measurement correctly identifies sample 1 as weakly water-wet. However, it suggests that samples 2 and 3 are oil-wet. The use of flat surfaces cannot capture the range of contact angle seen in reservoir rock which in turn has a very significant effect on remaining oil, presented in the next section.

### Remaining oil saturation

**Figure 5:**
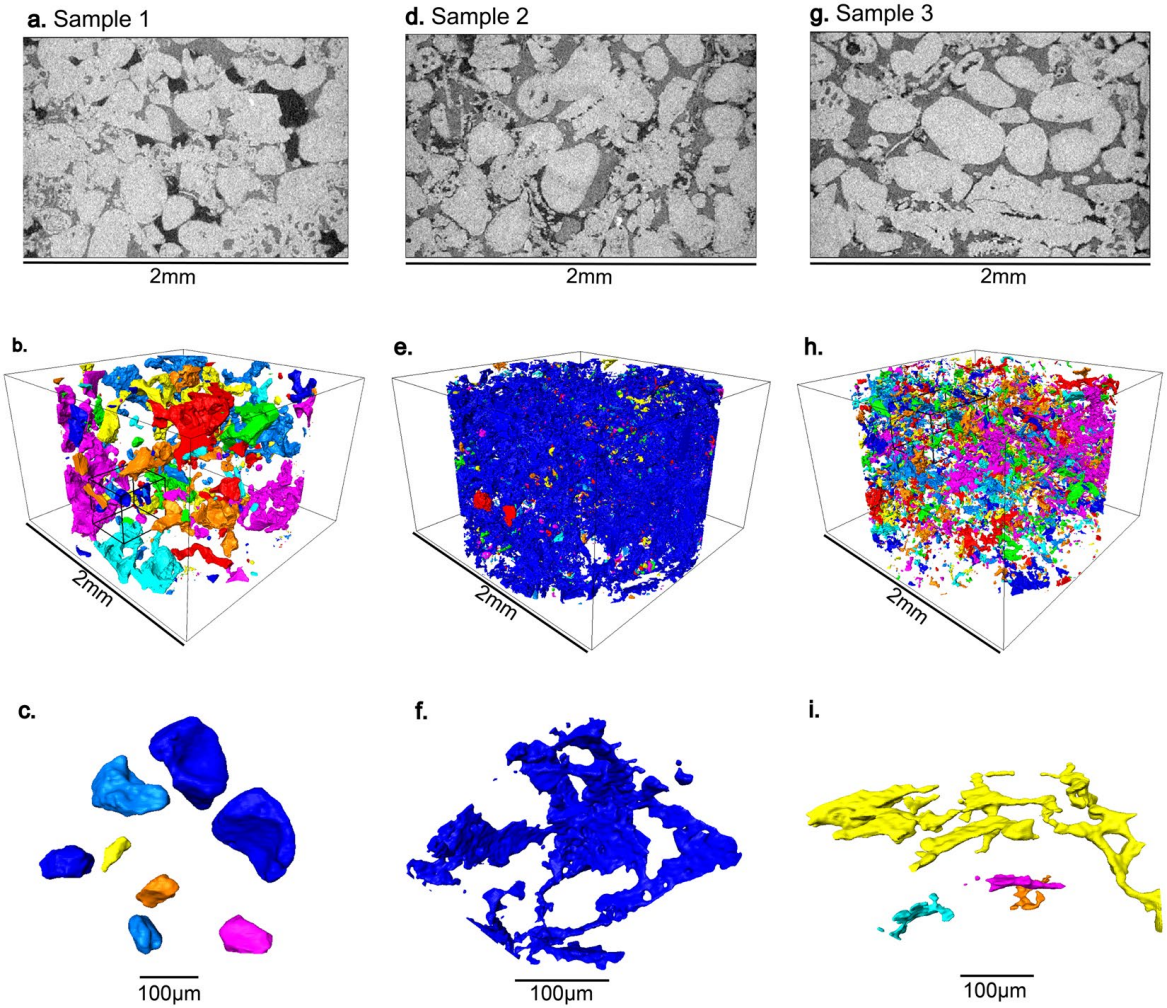
Two-dimensional cross-sections of three-dimensional X-ray micro-tomographic images (upper figures) and three-dimensional renderings of the remaining oil saturation after waterflooding (middle figures) with magnified images of oil ganglia (lower figures) that are highlighted in smaller black cubes within the larger images (middle figures). Samples 1, 2 and 3 are shown in (**a**,**b**,**c**), (**d**,**e**,**f**) and (**g**,**h**,**i**) respectively. The colors show different oil ganglia that appear trapped at the resolution of the image.

The remaining oil saturation was measured from segmented images after 20 pore volumes of brine waterflooding at a low flow rate, see Fig. 5. The capillary number defined as *μq/σ*, where *q* is the Darcy velocity of the injected brine, *μ* is the brine viscosity and *σ* is the interfacial tension between oil and brine, was estimated to be 6 × 10^−7^, 3 × 10^−7^ and 6 × 10^−7^ for samples 1, 2 and 3 respectively, representing capillary-controlled conditions^1^. In the weakly water-wet rock (sample 1), brine invaded pores as a wetting phase preferentially filling small pores and roughness, leaving oil in the middle of pores as a disconnected phase, which is similar to what was observed in earlier studies^27, 41–43^. The oil phase appears disconnected and is likely to represent the true residual, or minimum, saturation: further injection of water is unlikely to yield any further recovery. The disconnected oil forms approximately spherical shapes to minimize the surface area and thus minimizing surface free energy as shown in Fig. 5; the oil/brine surface area divided by the total volume was 3.02 × 10^−3^ *μ*m^−1^.

In the mixed-wet cases (samples 2 and 3), brine invaded the center of pores and left oil in small pores and corners. The remaining oil has a rough sheet-like structure Fig. 5, as seen in previous studies of oil-wet media^27, 41^. The mixed-wet cases had higher total oil/brine surface area to volume ratios of 8.52 × 10^−3^ *μ*m^−1^ and 4.26 × 10^−3^ *μ*m^−1^ for samples 2 and 3 respectively because the oil is principally confined to layers throughout the pore space^27^. The oil/brine surface area per unit volume of the more oil-wet sample (sample 2) is about three times greater than the weakly water-wet (sample 1), similar to the results of ref. 27. For sample 3, with the highest oil recovery, the remaining oil mainly fills the smaller pores, and appears disconnected at the resolution of the scan. In contrast, the oil phase in sample 2, which has the lowest recovery, still appears to be largely connected in oil layers: these layers, however, only allow very slow flow and do not yield good recovery for economic amounts of water injection (a few pore volumes).

**Figure 6:**
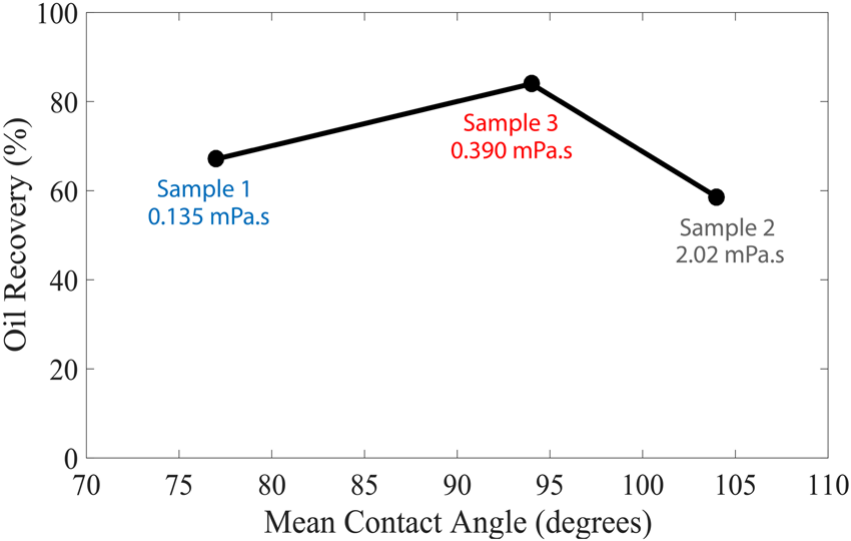
Oil recovery as a function of mean *in situ* contact angle. The oil viscosity is also shown.

The oil recovery is found to be a strong function of wettability and oil viscosity, Fig. 6. The ratio of oil to brine viscosity for samples 1, 2 and 3 are 0.289, 5.64 and 0.834 respectively. However, we find that recovery is not simply inversely related to viscosity ratio: the highest recovery is seen in sample 3 with an intermediate viscosity ratio, indicating that wettability also plays a key role in the behavior. The weakly water-wet rock (sample 1) showed a low oil recovery of 67.1% (remaining oil saturation of 32.9%). The maximum oil recovery of 84.0% (remaining oil 16.0%) was achieved by the mixed-wet rock (sample 3) with a mean contact angle close to 90° even though the oil viscosity is higher than in sample 1. The rapid deterioration in recovery was for the more oil-wet media (sample 2) with a substantially more viscous oil to 58.6% (remaining oil 41.4%). Core-scale studies on sandstones^44^ have shown that optimal recovery was obtained for a rock that appeared neither strongly water-wet nor oil-wet. However, only the core-scale wettability index was measured, and the behavior in terms of the pore-scale distribution of contact angle could not be assessed. At the pore scale, optimal recovery is found when the rock is not strongly water-wet, suppressing snap-off in small pores, which can trap oil, yet not so oil-wet as to confine all the oil to layers, which flow too slowly to provide significant recovery (1): as evident in Fig. 5, the lowest remaining saturation is seen when the oil appears confined to only some of the smaller pores.

### Primary cause of wettability alteration

**Figure 7:**
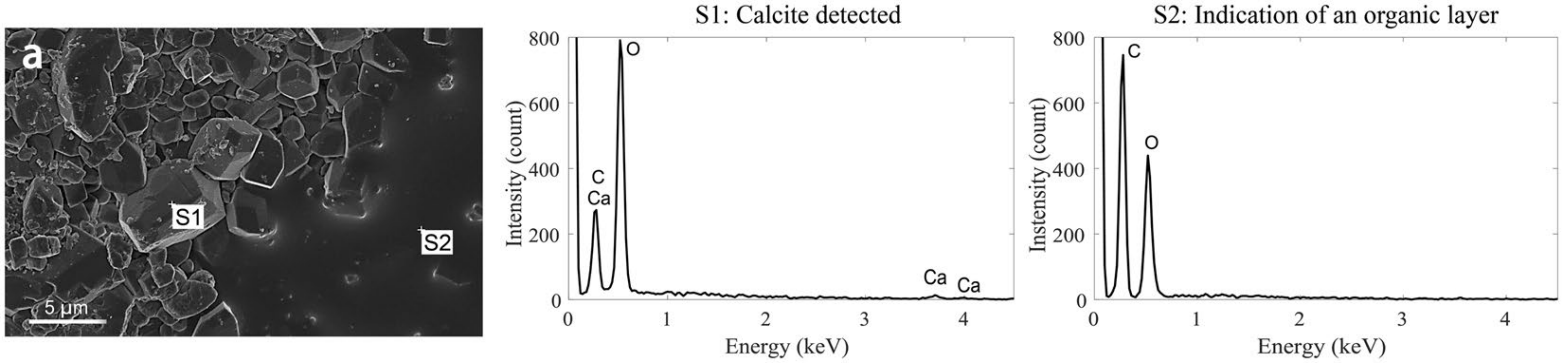
An SEM image of a pore that was filled with crude oil after drainage showing two distinctive surfaces at S1 and S2 where EDX analysis was conducted. The elemental analysis at location S1 shows calcium, carbon and oxygen indicating calcite. In contrast, the elemental analysis at location S2 shows a high peak of carbon with no calcium indicating the presence of an adsorbed organic layer.

The hypothesized cause of wettability alteration by the adsorption of organic layers on rock surfaces that are in direct contact with crude oil was investigated by scanning electron microscopy (SEM) and Energy Dispersive X-Ray analysis (EDX). Figure 7 shows a magnified view of location (A) in Fig. S4 in the Supplementary Information, which is a pore that was filled with crude oil after drainage and which could have an adsorbed organic layer. Two distinctive surfaces were observed in Fig. 7 and EDX analysis was conducted at both. Firstly, EDX was applied at location S1 at an uncoated grain surface and at a second location S2 with an adsorbed organic layer. Ca, C and O_2_ were detected at S1 indicating calcite which agrees with XRD analysis showing that 96% of this sample is calcite, Table S2. On the other hand, a large peak of carbon, C, with no Ca is observed at S2 which indicates the presence of an organic layer causing wettability alteration from water-wet towards oil-wet conditions. Please refer to the Supplementary Information for a detailed description of the sample preparation prior to SEM scan acquisition.

## Conclusions

We have presented a procedure to quantify *in situ* wettability using X-ray micro-CT imaging at subsurface conditions using crude oil to saturate carbonate rock extracted from a producing hydrocarbon reservoir. The *in situ* wettability was quantified and a range of contact angle both above and below 90° was measured for rocks with a uniform, calcite, mineralogy. Pores filled with brine remained water-wet. On the other hand, pores filled with crude oil could become oil-wet with contact angles up to around 140°, consistent with measurements on flat mineral surfaces. We investigated the main cause of wettability alteration and showed that there is an adsorbed organic layer in some parts of pores which were filled with crude oil after drainage by using SEM and EDX analysis. However, some parts of pores that were filled with crude oil also remained water-wet, which suggests the possible presence of stable water layers within the rock surface roughness preventing direct contact between crude oil and the solid surface. Oil recovery, after waterflooding, was highest for the mixed-wet sample with an average contact angle close to 90°. While it is considered that controlled salinity waterflooding should be designed to drive the rock to more water-wet conditions^16^, these results suggest that mixed-wettability instead gives the most favorable recoveries, where the oil remains connected in layers but the water conductance is low^1^.

In the future, the distribution of *in situ* contact angles could be input into pore-scale models to predict multiphase flow properties and to provide a pore-by-pore comparison of predicted and imaged fluid distributions. More generally, such methods provide a compelling methodology for assessing local displacement efficiency and can aid the design of injection brine composition to maximize recovery. This assessment of wettability can also be used to evaluate carbon dioxide storage security, particularly in depleted hydrocarbon fields where the rock may have undergone a wettability alteration.

## Materials and Methods

### Imaging fluid distributions using X-ray micro-CT

We used a ZEISS Xradia VersaXRM-500 X-ray micro-CT scanner to acquire high resolution three-dimensional images of reservoir rocks and the fluids within them. CO_2_ was injected in clean and dry representative mini-samples to displace air followed by brine injection to fully saturate the rock. Then, subsurface conditions were established (60 °C or 80 °C and 10 MPa) and primary drainage (crude oil injection) was performed, followed by aging over three weeks to restore rock wettability. Finally, 20 pore volumes of brine were injected at 15 *μ*L/min or 10 *μ*L/min. Fluids were allowed to reach equilibrium for two hours before acquiring 2 *μ*m/voxel scans to characterize *in situ* wettability and measure fluid saturations. A detailed description of the experimental method is provided in the Supplementary Information.

### Rock and fluid properties

Three cores of diameter 4.8 mm and length between 13 and 16 mm from a multi-billion barrel producing reservoir in the Middle East were selected: they are principally composed of calcite (96.5 ± 1.9 weight%). We have used a light crude oil (from the same reservoir, surface-condition density of 830 kg/m^3^) and a heavier crude oil (Arabian Medium, density of 870 kg/m^3^) referred to as crude oil A and crude oil B respectively. Potassium iodide (KI) brine solution was used to obtain effective phase contrast^45^. For detailed descriptions of the fluids used please refer to the Supplementary Information.

### Image segmentation

All images were segmented into three phases (oil, brine, rock) from the raw X-ray micro-CT images using a machine learning-based image segmentation known as Trainable WEKA Segmentation^46^, which applies a feature-based segmentation. Fast-random algorithm and texture filters (mean and variance) were chosen and pixels from each phase (oil, brine and rock) were annotated manually to train a classifier model. The trained classifier model recognizes characteristic shapes, facilitating the measurement of contact angle, and was used to segment the images, Fig. S7. Detailed descriptions of the image processing and segmentation can be found in the Supplementary Information.

### Contact angle measurement

**Figure 8:**
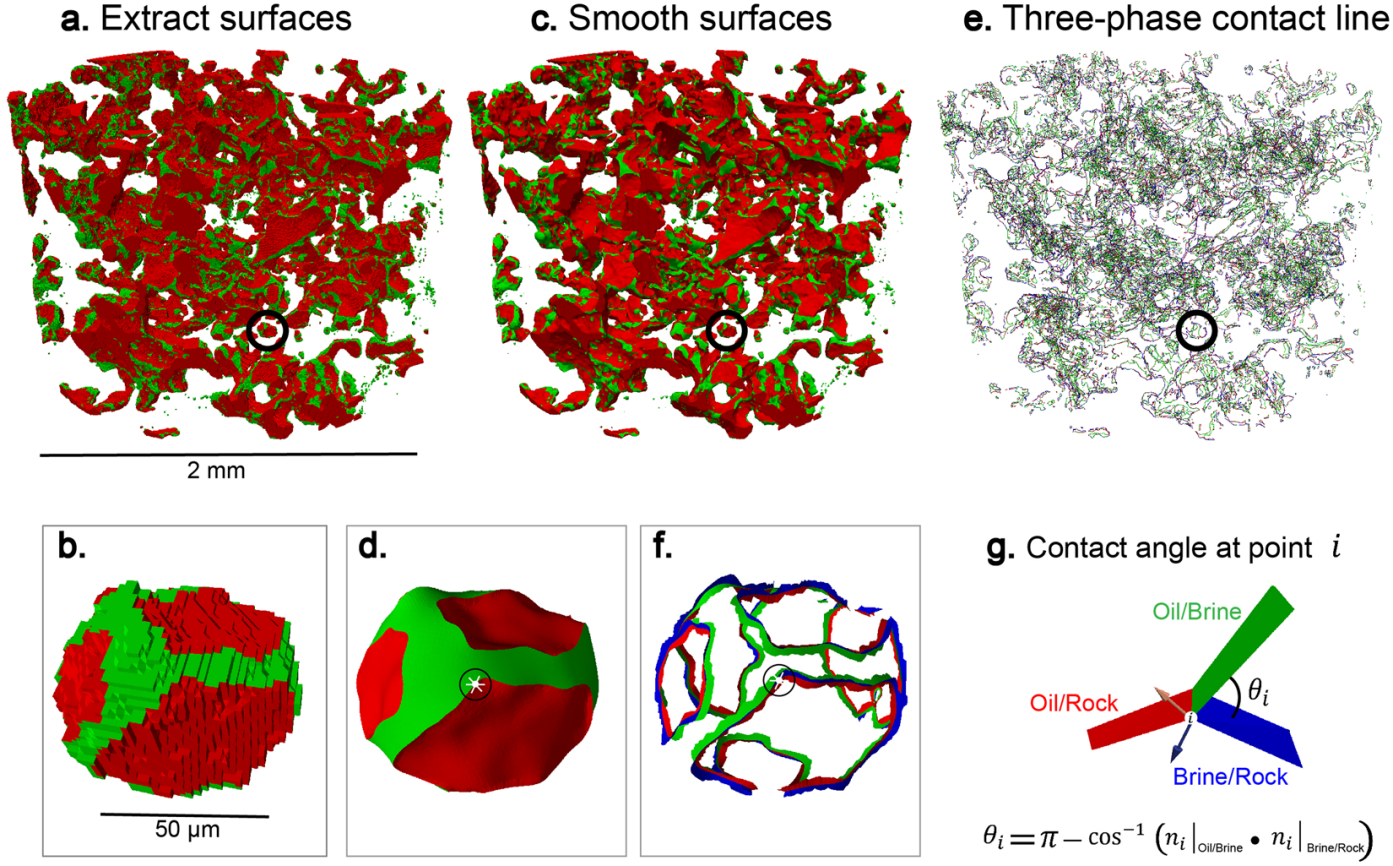
Automatic *in situ* contact angle measurement workflow demonstrated on oil ganglia in Sample 1 with a magnified view of a single trapped oil ganglion highlighted in the black circle. Rock and brine were rendered transparent. (**a**,**b**) Extracted oil-brine surface (green) and oil-rock surface (red). (**c**,**d**) Smoothed surfaces with a selected example point indicated by the white dot. (**e**,**f**) Three-phase contact line with oil-brine surface (green), oil-rock surface (red) and brine-rock surface (blue) with the same selected example point highlighted by the back circle. (**g**) Contact angle measurement of 52° at the selected point from the vector dot product of the oil-brine interface and rock interface vectors.

The automated algorithm developed by ref. 32 measures the *in situ* contact angle distribution between immiscible fluids from segmented images. This method fits a smoothed constant-curvature surface to the fluid/fluid interface and records its intersection with the solid. This approach removes the subjectivity associated with the manual method, while providing many hundreds of thousands of measurements throughout the sample. Figure 8 illustrates how the measurements are made (images from Paraview software): firstly, fluid/fluid and fluid/solid surfaces are identified and meshed; secondly, the Gaussian smoothed surfaces are generated to remove artefacts from voxelization; thirdly the surface is deformed to impose a more constant curvature on the fluid/fluid surfaces, consistent with capillary equilibrium; and, finally, perpendicular vectors to the mesh are defined along the three-phase contact line and the contact angle is determined by the vector dot product of vectors representing the oil-brine interface and the rock interface at the three-phase contact line.

The manual method developed by ref. 26 was used to measure contact angle at a few selected points for comparison purposes. First of all, a three-phase contact line is extracted from the segmented image. Then a point is selected for measurement for which a slice of the image is taken orthogonal to the three-phase contact line at the point chosen. Finally, the contact angle is measured on the perpendicular slice through brine by eye. The workflow, followed to measure contact angles manually at the same locations, is shown in Fig. S6.

Before measuring the *in situ* contact angle distribution by the automatic method^32^ for the whole imaged volume, a small sub-volume was extracted from each sample, shown in Fig. S5, for which contact angle was measured by hand manually^26^ and automatically^32^ at the same locations as illustrated in Fig. S6 (see the Materials and Methods section for how this is done). Figure S11 shows good agreement between the manual and automatic methods, allowing us to use the automatic algorithm confidently for the entire dataset.

### Data Availability

The datasets that support the findings of this study are available from [Abu Dhabi National Oil Company, ADNOC] but restrictions apply to the availability of these datasets, which were used under license for the current study, and so are not publicly available. Datasets are however available from the authors upon request and with permission of [ADNOC].

## Electronic supplementary material

Supplementary Information
