# Supplementary material for: In situ characterization of mixed-wettability in a reservoir rock at subsurface conditions
Source: Sci Rep. 2017 Sep 7;7:10753. doi: 10.1038/s41598-017-10992-w (PMC5589931; doi:10.1038/s41598-017-10992-w)
Supplement: Supplementary file 1 — Supplementary Information [file 41598_2017_10992_MOESM1_ESM.pdf]

In situ characterization of mixed-wettability in a reservoir rock at subsurface conditions

Amer M. Alhammadi[1], Ahmed AlRatrout[1], Kamaljit Singh[1], Branko Bijeljic[1], and Martin J. Blunt*[1]

[1]Department of Earth Science and Engineering, Imperial College London, London, SW7 2AZ
*m.blunt@imperial.ac.uk

Supplementary Information

Fluid and rock properties

Two crude oils were used, crude oil A (a light oil from the same reservoir as the rock samples) and crude oil B (Arabian Medium) which is heavier: see Table S1 for an analysis of their composition performed by Weatherford Labs to measure the content of the heavier fraction contents in each crude oil, including resins and asphaltenes that contain polar components able to alter the rock wettability.

Table S1. Crude oil A and B properties

	Crude oil A	Crude oil B
Density at 21°C, kg/m^3	830 ± 5	870 ± 5
Saturates, wt%	55.25	33.54
Aromatics, wt%	38.07	52.88
Resins, wt%	6.22	9.30
Asphaltenes, wt%	0.46	4.28
Total Acid Number, mg KOH/g	0.24	<0.1
Total Base Number, ppm	356	124

A brine solution was prepared using 7 weight percent of potassium iodide (KI) (purity ≥ 99.0%, Sigma-Aldarich, UK) mixed with deionized water. This brine can be distinguished from oil and rock phases for effective image segmentation[1]. The brine had a density of 1052.1 ± 2.2 kg/m^3[2] and a pH value of 6.91 ± 0.2 measured with FiveGo pH meter, Mettler Toledo.

Three mini-samples were used in this study and were drilled from a larger rock sample extracted from a very large producing carbonate hydrocarbon reservoir which is mainly calcite (96.5% ± 1.9%), Table S2. The phase identification and quantification using X-ray diffraction (XRD) was performed at the Natural History Museum, London, UK. The XRD analysis were conducted on 5 grams of crushed rock taken from locations close to the drilled mini-samples. The larger core plug has a total helium porosity of 27.0 % and a permeability of 6.8×10^{-13} m^2 (686 mD). The porosity and pore volume of the used mini-samples are shown in Table S3. We did not measure the helium porosity on mini-sample 1, so assumed the overall value 27.0%.

Table S2. Mineralogical composition

Phase	Calcite	Dolomite	Kaolinite	Quartz
Phase proportion, wt%	96.5	1.5	1.1	0.8
Estimated error, wt%	1.9	0.3	0.2	0.4

Table S3. Properties of the samples used in this study

Samples	1	2	3
1. Diameter, mm	4.75	4.75	4.75
2. Length, mm	16.3	13.1	13.6
3. Helium Porosity, %	27.0	31.7	30.5
4. Segmented porosity, %	20.8	20.4	20.2
5. Pore volume, μL	78	74	74

Extracting representative samples

Carbonate reservoir rocks are made up of complex pore geometry with a wide range of size. To extract representative mini-samples, the whole core plug was dry scanned at 40 μm/voxel and drilling locations were selected to avoid large impermeable minerals and large pores, Fig. S1.

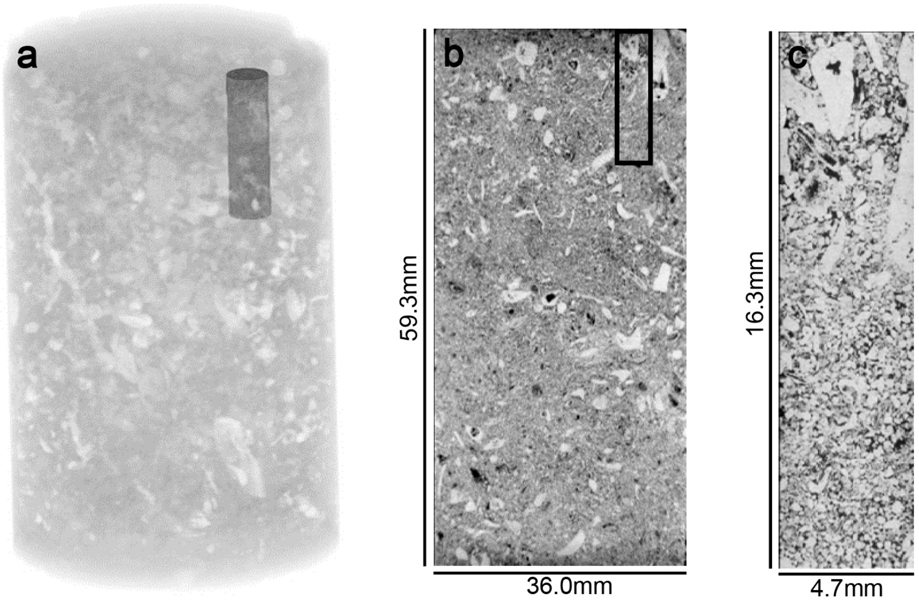

Figure S1. a. Semi-transparent 3D rendering of the whole core plug showing the location of mini-sample 1 in dark gray. b. Cross-sectional view of the whole core plug showing rock heterogeneity and the location of mini-sample 1. c. Cross-sectional view of a higher resolution dry scan (5.5 μm/voxel) of mini-sample 1.

Experimental apparatus and flow procedure

An Xradia VersaXRM-500 X-ray micro-CT was used to acquire high resolution three-dimensional images to characterize *in situ* wettability at sub-surface conditions. Contrast scans were taken before each flow experiment to ensure an effective image contrast between phases (oil, brine and rock). The apparatus used to elevate system pressure and control flow rate is shown in Fig. S2. The pumps are connected to a flow cell, which is magnified on the right in Fig. S2, placed inside the X-ray enclosure and connected to a PID controller to control temperature. The flow cell is illustrated with three-dimensional cross-sectional view of the internal components.

First of all, a dried mini-sample is placed inside a Viton rubber sleeve (Cole-Parmer, UK) and connected to flexible 1/16 inch PEEK tubing (Kinesis Ltd., UK) from both sides through custom made stainless steel end fittings. The rock sample connected to PEEK tubing is inserted carefully into the middle of a carbon fiber sleeve, transparent to X-rays[1], and connected to flow cell stainless steel metal end pieces. Flexible PEEK tubing was used to allow the flow cell to rotate freely 360 degrees during scan acquisition. A flexible heating jacket (Omega, UK) was wrapped around the carbon fiber sleeve to elevate temperature, which was measured by a thermocouple (Omega, UK) placed next to the sample and controlled by a PID controller. High pressure Teledyne Isco syringe pumps (Teledyne Isco, US) were used for fluid injection and to maintain pressure. A pressure of 1.5 MPa using deionized water was applied in the confining isolated space between the Viton sleeve and the carbon fiber sleeve as shown in Fig. S2 and a full dry scan was acquired.

Then, CO_2 was injected to displace air for an hour followed by brine injection using a Teledyne syringe pump Model 100D for another hour at 0.3 mL/min to displace all the CO_2 and fully saturate the rock sample. Temperature and pressure were elevated to 60°C or 80°C and 10 MPa with a confining pressure of 11.5 MPa. Twenty pore volumes of oil were injected at 15 μL/min or 10 μL/min to reach connate water saturation and mimic the invasion of crude oil after migration from source rock to reservoir formations. Oil invades the center of larger pores as the non-wetting phase under a capillary dominated flow regime and the formation brine remains in small pores, pore corners and crevices due to capillary forces[3]. Then, the system was allowed to reach equilibrium for two hours before acquiring the scans. 5.5 μm/voxel scans were acquired for the sample when dry and after oil injection in mini-sample 3 to be used in the SEM study. To restore reservoir wettability, three aging procedures

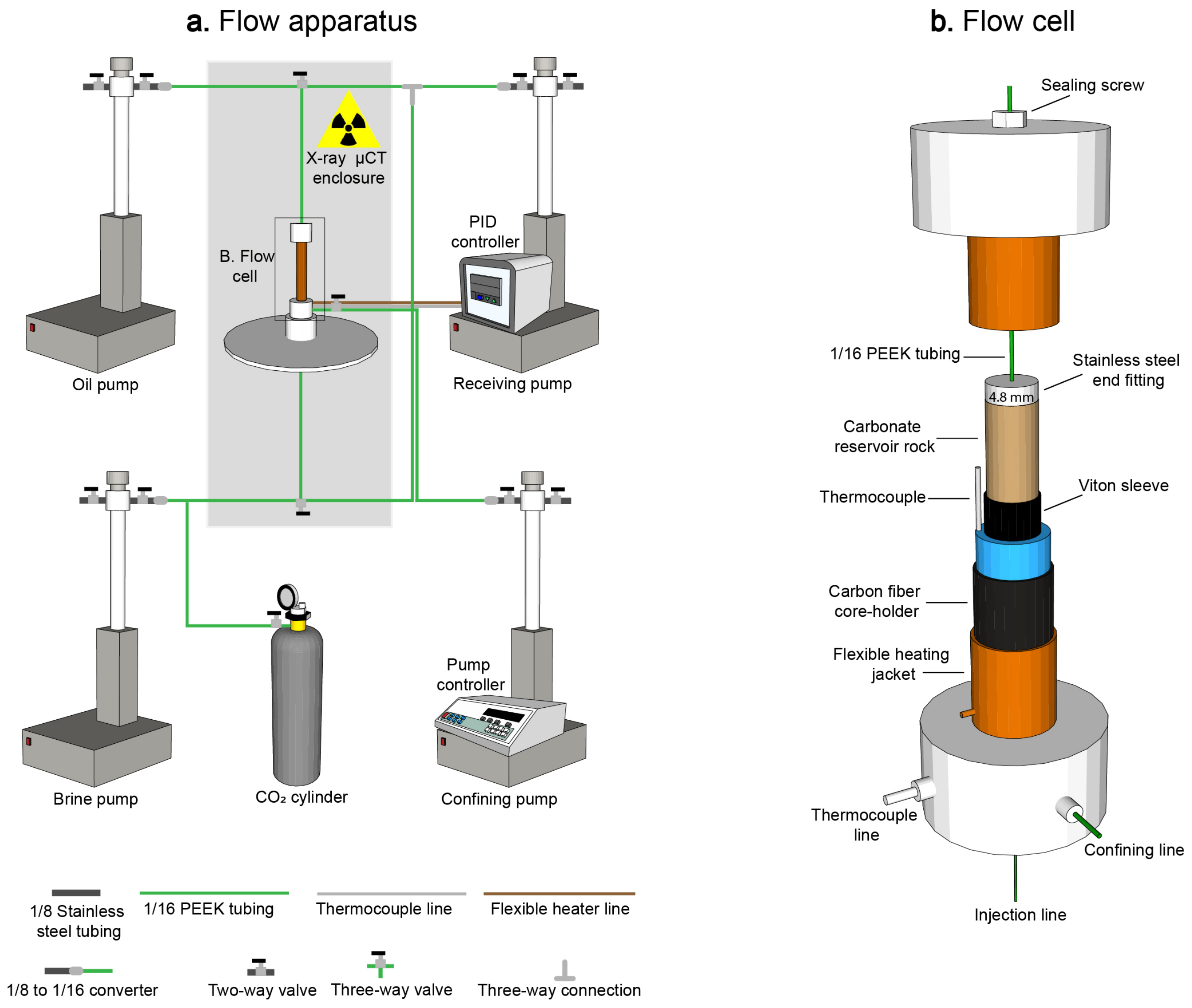

Figure S2. a. Experimental apparatus with pump combination, CO_2 source and PID controller connected to the flow cell inside an X-ray enclosure. b. Detailed description of the flow cell showing the cross-sectional view from inside.

were performed as described in the following section. During the aging process, polar components present in high molecular crude oil fractions, such as asphaltene, interact with mineral surfaces resulting in the adsorption of a layer of asphaltene and other crude oil polar fractions[4,5] and a change of wettability of rock surfaces in direct contact with crude oil towards a more oil-wet state takes place[5–8]. The aging process was followed by waterflooding of 20 pore volumes of brine (7 weight % KI) to obtain the remaining oil saturation. Again the system was left for two hours to reach equilibrium and then high resolution (2 μm/voxel) scans were acquired at 60°C and 10 MPa from which the *in situ* contact angle distribution and fluid saturation were measured.

Aging protocols

To restore reservoir wettability and investigate its impact on remaining oil saturation, three aging protocols were employed using raw crude oil and crude oil-heptane mixtures at different conditions. Heptane (purity 99%, Sigma-Aldarich, UK) was used to induce asphaltene precipitation. Asphaltene precipitation is directly controlled by crude oil solvency towards its asphaltene which is influenced by crude oil composition, temperature and pressure[9]. The amount of added heptane was close to and at the onset value of asphaltene precipitation[10–12]. For crude oil A, 90% of heptane was added which is slightly higher than the asphaltene onset value of precipitation (87.8% heptane and 12.2% crude oil A) to precipitate almost all the available asphaltenes.

For crude oil B, used by[10], the same onset value of precipitation was used (72% of heptane and 28% of crude oil B) to alter rock wettability and at same time prevent any reduction in permeability[10]. The viscosities of the oil phase at experimental temperature and ambient pressure are shown in Table S4. Values at 60 °C were calculated from the linear relationship between the natural logarithm of viscosity and inverse of temperature from viscosity measurements at 25°C, 30°C, 35°C, 40°C, 55°C, 70°C and 80°C, shown in Fig. S3. The viscosity was measured using an Anton Paar MCR301 rheometer.

Table S4. Viscosity of oil phases used in the three experiments

Experiment	Viscosity, mPa.s	Temperature, °C
1. 10% crude oil A & 90% heptane	0.135	60
2. 100% crude oil A	2.02	80
3. 28% crude oil B & 72% heptane	0.390	60

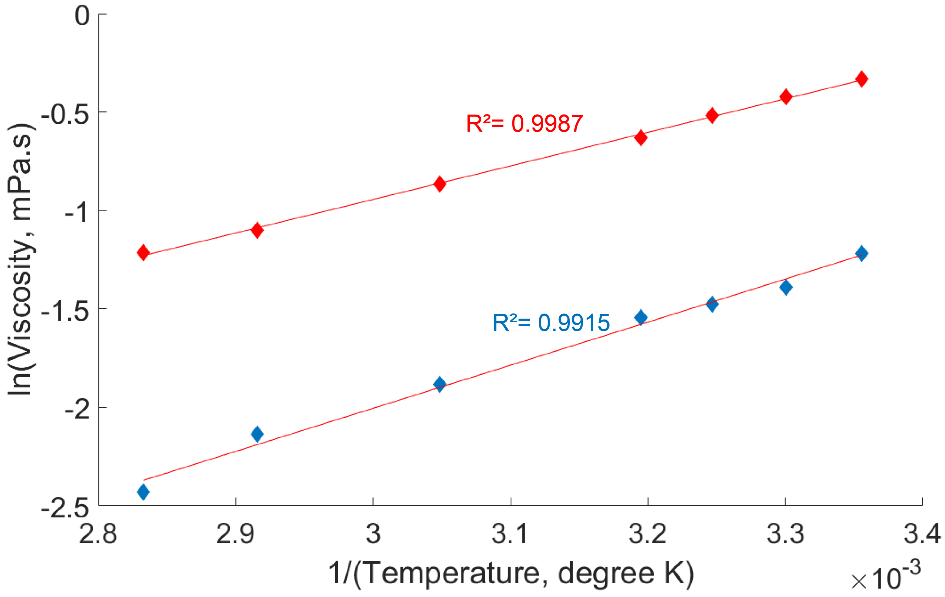

Figure S3. Linear relationship between inverse of temperature (K) and natural logarithm of viscosity (mPas). 10% crude oil A and 90 % heptane is shown in blue and 28% crude oil B and 72% heptane is shown in red.

Mini-sample 1 was statically aged for three weeks at 60°C and 10 MPa after primary drainage of 20 pore volumes of the crude oil A-heptane mixture at 15μL/min. Mini-sample 2 was dynamically aged for one week and statically aged for two weeks at 80°C and 10 MPa after primary drainage of 20 pore volumes of the raw crude oil A at 10 μL/min. Mini-sample 3 was dynamically aged for three weeks at 60°C and 10 MPa after primary drainage of 20 pore volumes of the crude oil B-heptane mixture at 15μL/min. Dynamic aging means a continuous injection of the oil phase while static aging means no flow. Dynamic aging speeds up wettability alternation[13].

Asphaltene layer observation and identification

A high resolution field emission gun scanning electron microscope (LEO Gemini 1525 FEGSEM) was used to study sample 3. Sample 3 was cleaned and prepared after completing the flow experiment by following similar procedure to Idowu et al.[14]. Remaining crude oil was removed by injecting decalin followed by heptane. Decalin was used as a buffer phase between crude oil and heptane to avoid any further asphaltene precipitation[13]. Then, most of the heptane was removed by waterflooding and the sample was dried before being cut crosswise carefully with a Struers saw using water as a coolant. The sample was then soaked in methanol to remove any remaining salt followed by drying and chromium coating before acquiring SEM scans. The full cut face was first scanned at a lower resolution of (9.5 μm/pixel) as shown in Fig. S4. Then higher resolutions scans were acquired at three locations of interest A, B and C. The same cross-sectional slice was found manually from the micro-CT image after drainage on the right, Fig. S4. An intact area of interest, highlighted by the black square, was selected for this study and

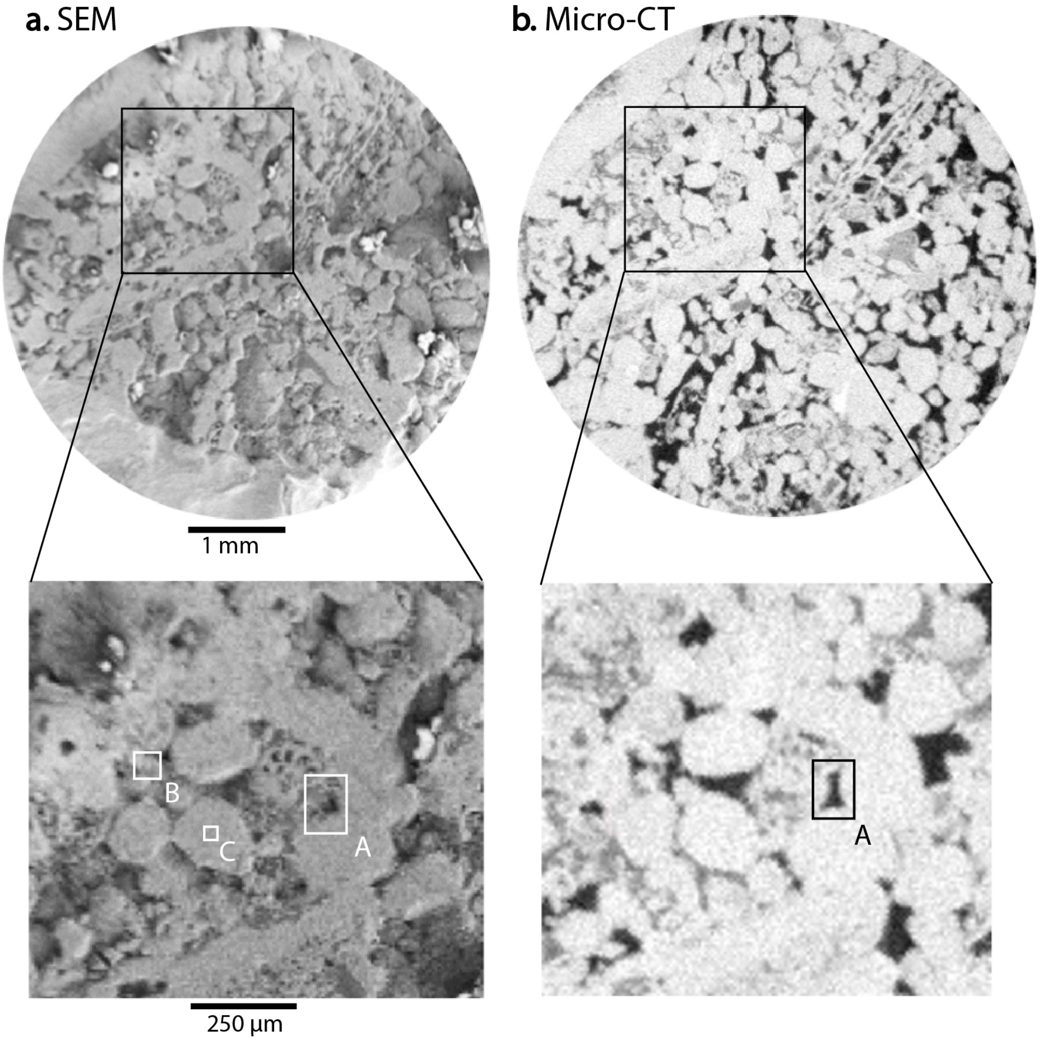

Figure S4. a. Full cut face of cleaned mini-sample 3 taken by SEM with an area of interest highlighted by the black square including three investigated locations highlighted in the lower figure in white squares (A, B and C) to investigate the adsorbed organic layer, and rock surface roughness at the micro-scale and the nano-scale respectively. b. The same slice found manually from a 3D micro-CT image after drainage showing a selected pore filled with oil.

the three locations were investigated. Location (A) was selected to investigate the presence of an adsorbed organic layer in a pore that was filled with crude oil during primary drainage. Energy Dispersive X-Ray (EDX) analysis was conducted at an energy of 2.5 keV to identify what could be an adsorbed organic layer at location (A). Locations B and C were used to study rock surface roughness at the micron and at the nano scales.

Workflow for comparison of manual and automatic measurements

A small sub-volume was extracted from each sample to compare manual and automatic contact angle measurements. Fig. S5 shows the sub-volumes selected with raw images at the top and segmented images at the base. To measure contact angle manually at the same location as the automatic measurements, the the steps illustrated in Fig. S6 were followed. Firstly, Paraview software was used to render the generated smoothed surfaces of brine (blue) and oil (red) by the automatic method in 3D; then x, y and z coordinates of a selected automatic point was identified spatially as shown by the yellow point in Fig. S6. Then, Avizo software was used to render rock as transparent and brine (blue) and oil (red) and the same point was identified manually and a cross-sectional slice was placed at the yellow point as shown in the middle figure. Finally, the cross-sectional slice was rotated to be orthogonal to the three-phase contact line and the contact angle is measured as shown in the right figure. A non-local means filter[15,16] was used to remove noise in the images used in the manual method only where the contact angle was measured by eye.

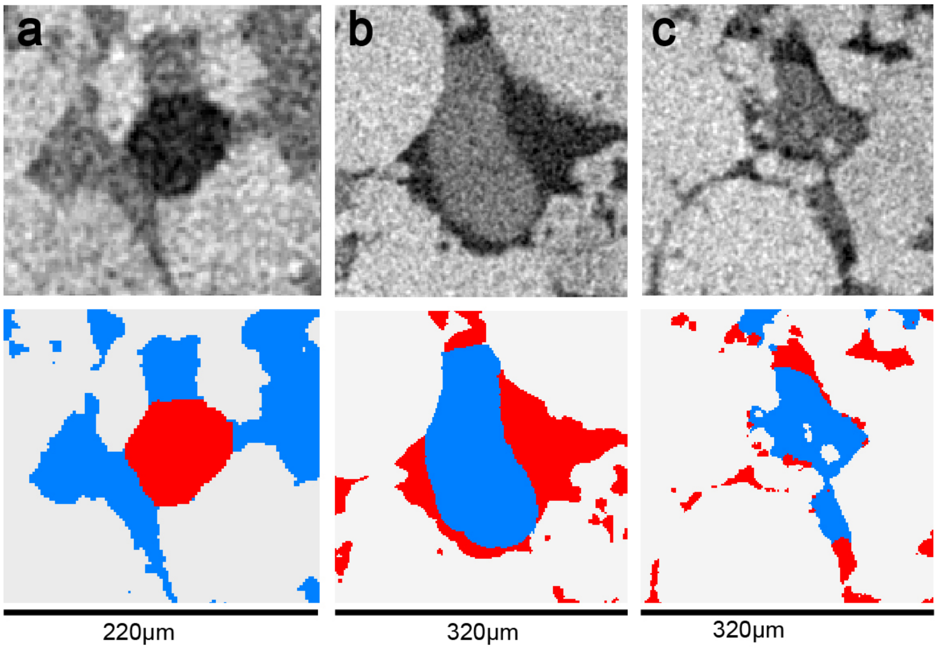

Figure S5. Three small sub-volumes extracted from the reservoir rock samples. Top row shows a cross-sectional view of raw X-ray images and the bottom row shows the same but segmented images. The oil, brine and rock phases are in black, dark gray and light gray in the raw X-ray images and in red, blue and white in the segmented images respectively. The sub-volumes from samples 1, 2 and 3 are shown in a, b and c respectively.

Image acquisition, processing and segmentation

All scans were acquired using the Xradia VersaXRM-500 X-ray micro-CT. Energy and power of 80 keV and 7 W were applied with a sample rotation of 360 degrees. No source filter was used. For scans at 5.5 μm/voxel, an objective of 0.4X was used with an exposure time of 1.5 seconds and a total of 2,000 projections. For high resolution scans at 2.0 μm/voxel, an objective of 4X was used with an exposure time of 3.5 seconds and 5,000 projections. The X-ray radiation intensity was 5,000-10,000 counts/second. The tomograms were reconstructed using Zeiss Reconstructor Software and an appropriate center shift correction value was applied for each scan. All image processing was conducted using Avizo 9.0.1 Software (FEI Company, Hillsboro, Oregon, US) and Fiji (ImageJ). A machine learning-based image segmentation plug-in in Fiji (ImageJ) software known as Trainable WEKA Segmentation (TWS) was used to apply feature-based segmentation on the raw gray-scale micro-CT images[17]. No noise reduction filter was applied prior to segmentation to avoid averaging of voxel values especially at the thee-phase contact line at which contact angle is measured. Fast-random algorithm and texture filters (mean and variance) were chosen and pixels from oil, brine and rock were selected manually to train a classifier model. Then, the trained classifier model was applied to segment the three phases (oil, brine and rock) from the raw micro-CT images, Fig. S7. The selected random-forest algorithm has been used in biomedical applications, such as segmenting membranes from electron microscopy images[18], segmenting vessels from X-ray computed tomography images[19] and lung tissue[20]. Furthermore, it has been used in minerals engineering applications to segment packed particle bed samples[21].

The impact of segmentation accuracy on contact angle distribution

The automated algorithm is applied on segmented three-dimensional images, which means that segmentation accuracy is crucial to obtain valid contact angle distribution. Therefore, in this subsection a sensitivity analysis was performed on sample 2 (mixed-wet with more oil-wet surfaces) that is the most challenging case, containing oil ganglia in very small pores and crevices. Preserving the right shape of the fluid-fluid interface at these small pores and crevices is challenging. In this study, six different combinations of image filtering and segmentation were performed as shown in Fig. S8.

First of all, the raw image was segmented without applying any noise filter to avoid averaging of voxel values especially at the thee-phase contact line at which contact angle is measured. The first method was by Trainable WEKA Segmentation (TWS)[17] discussed earlier. The distribution of contact angle had a mean value of 104°±25.8° with a total of 1.41 million values as shown in Table S5 and in gray in Fig. S10. The same distribution was presented in the main paper. The comparison study, between the randomly selected points from the automated algorithm to that measured at the same locations by the manual

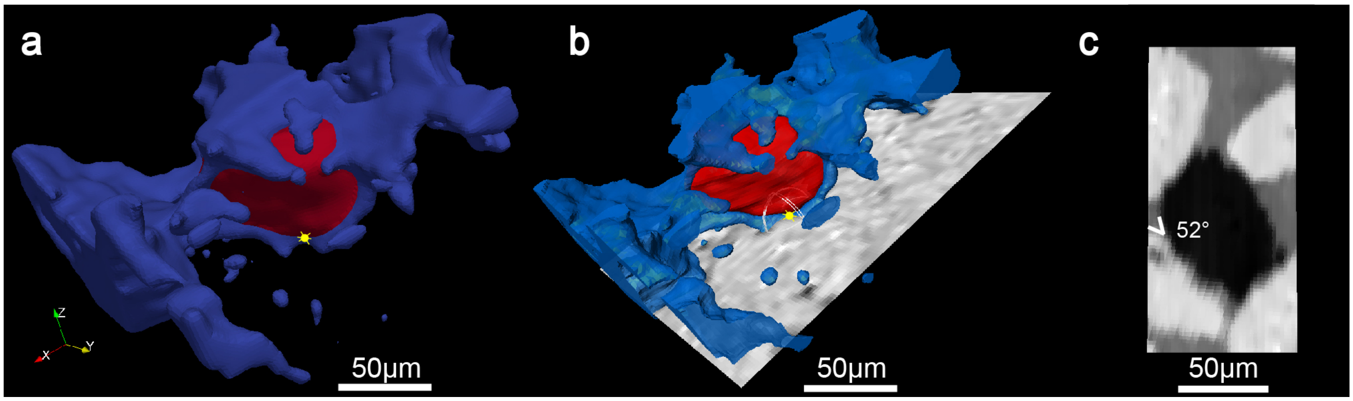

Figure S6. a. The x, y and z coordinates of an automatically measured point (with an estimated contact angle of 50°) was used to locate the point spatially on the generated smoothed brine and oil surfaces using the automatic method in blue and red respectively by using Paraview software. The located point is shown as the yellow dot. b. Avizo software was used to locate the same point manually and conduct the manual measurement at the same location. For illustrative purposes the brine is shown in blue, the oil in red, while the rock is transparent. The same point was identified manually and the the filtered slice was placed at the point to measure the manual contact angle by eye as shown in b. Finally, the slice was rotated to be orthogonal to the three-phase contact line and contact angle was measured by eye showing value of 52° in this case, compared to the automatic value of 50°.

method, confirms that this distribution is representative, as shown in Fig. S11. The second method was watershed by which oil, brine and rock phases were segmented with 2D-histogram segmentation that depends on the gradient magnitude and the image intensity histogram to generate representative seeds and then applies a watershed algorithm to label all voxels[22]. However, the raw images contains significant amounts of noise, so segmenting the raw image with watershed segmentation and without applying any noise filter resulted in a poor segmentation as shown in Fig. S8 and Fig. S9 part C where one phase frequently appears as a small feature surrounded by the other phases. The distribution from such segmentation is non-representative, even though it might contain partially correct measurements. The mean contact angle was less than 90° and the distribution has the largest number of values, 3.72 million and the widest standard deviation of 29.0°.

The raw image was filtered by the non-local mean filter to remove noise and preserve edges[15,16]. The filtered image was then segmented by three-phase watershed, two-phase watershed and three-phase Trainable WEKA Segmentation. Three-phase means that segmentation was performed to segment all three phases together. Two-phase segmentation means that a dataset is segmented twice and one phase is applied as a mask, which provides more flexibility in acquiring better image segmentation. For example, the first segmentation is rock as phase 1 and the oil-brine as phase 2. The second segmentation is brine as phase 1 and oil-rock as phase 2. Finally, rock from the first segmentation is masked over the second segmentation to give the third phase which is oil.

The contact angle measured from the two-phase watershed segmentation after applying the non-local means filter generated a distribution with a mean contact angle of 98.5°±23.4° and 1.31 million counts shown in green in Fig. S10. Its mean contact angle value and and number of counts are the closest to the Trainable WEKA Segmentation on the raw image. However, there is a difference of 5.5 ° which is most likely due to voxel averaging especially close to the three-phase contact line resulting in the inversion of the fluid-fluid interface close to the three-phase contact generating lower contact angle values, as shown in Fig. S9. The issue of fluid-fluid interface inversion becomes even more severe for three-phase watershed segmentation, resulting in an even lower mean contact angle value of 87.8°±23.9°. Furthermore, the very small regions of oil in the corners becomes more difficult to be to be segmented and they are labeled as brine instead, as shown in Figure S9. The loss of these little oil regions results in a decrease in the contact angle counts (800 thousand) compared to the 1.41 million and 1.31 million from the previous segmentations. The three-phase Trainable WEKA Segmentation had similar issue to the three-phase watershed and had a mean contact angle lower than 90°. The three-phase watershed segmentation after applying an Anisotropic Diffusion filter had similar mean contact value (86.2°±20.6°) to that from the non-local means filter with three-phase watershed segmentation with a difference of only 1.6°.

In conclusion, the automated algorithm is able to find a representative contact angle distribution on images that are correctly segmented. A comparison study of contact angle measurement at randomly selected points from the automated algorithm compared to points measured manually at the same location is recommended to confirm the accuracy of the segmentation as in Fig. S11.

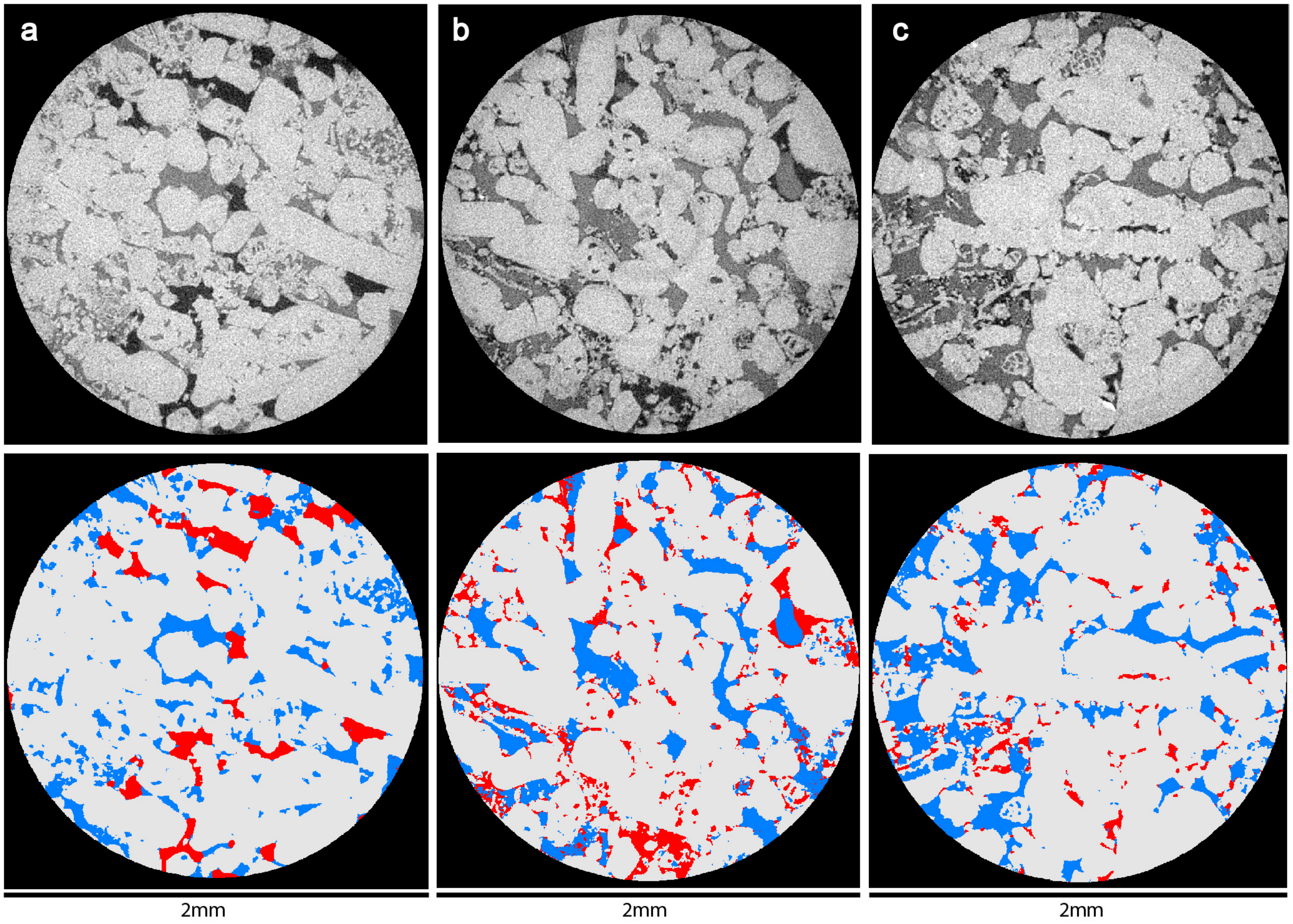

Figure S7. Two-dimensional slices of three-dimensional raw micro-CT images (top figures, a, b and c) for samples 1,2 and 3 respectively. Two-dimensional cross-sections of the segmented images are shown on the bottom row for the same slices with oil, brine and rock in red, blue and white respectively.

Contact angle measured on calcite mineral

Three flat calcite mineral crystals were aged at similar conditions to the micro-CT flow experiments using the same oils. The crystals were aged for three weeks at 60°C and 80°C and ambient pressure. A Ramé-Hart contact angle goniometer (Ramé-Hart, US) and DROPimage Advanced software were used to measure contact angle at ambient conditions. The captive drop method[23] was followed to measure the contact angle between oils and brine (7 weight % KI). A mean value of contact angle was obtained for each case on untreated and treated calcite crystals by taking 100 measurements on each side of the drop (one measurement per second).

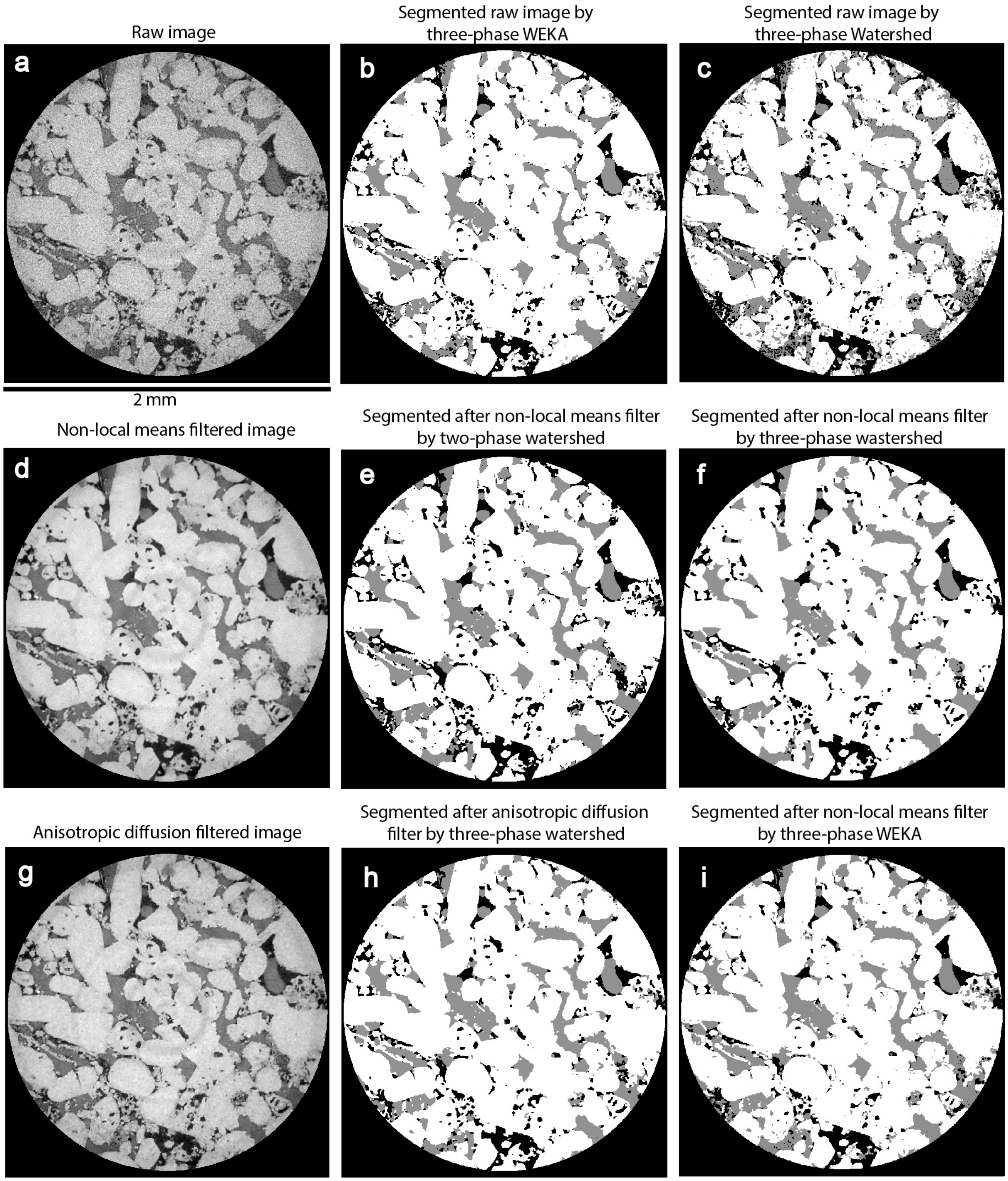

Figure S8. Six combinations of noise filter and segmentation options were analyzed. a. A raw two-dimensional cross-sectional image of sample 2 with oil, brine and rock in black, dark gray and light gray respectively. b. Segmented raw image using three-phase Trainable WEKA Segmentation. c. Segmented raw image using three-phase watershed segmentation. d. Filtered image using the non-local means filter. e. Segmented image by two-phase watershed segmentation after applying the non-local means filter. f. Segmented image by three-phase watershed segmentation after applying the non-local means filter. g.The image after applying an anisotropic diffusion filter. h. Segmented image by thee-phase watershed segmentation after applying the anisotropic diffusion filter. i. Segmented image using thee-phase Trainable WEKA Segmentation after applying the non-local means filter.

Table S5. Measured contact angles on images after applying different segmentation and filtering procedures.

Segmentation	Contact angle, °	Counts	Histogram color
1. Raw by three-phase watershed	86.0±29.0	3.72 million	blue
2. Anisotropic diffusion filter by three-phase watershed	86.2±20.6	1.11 million	yellow
3. Non-local means filter and two-phase watershed	98.5±23.4	1.31 million	green
4. Raw using three-phase Trainable WEKA	104±25.8	1.41 million	gray
5. Non-local means filter and three-phase watershed	87.8±23.9	800 thousand	red
6. Non-local means filter and three-phase Trainable WEKA	82.1±24.2	1.63 million	brown

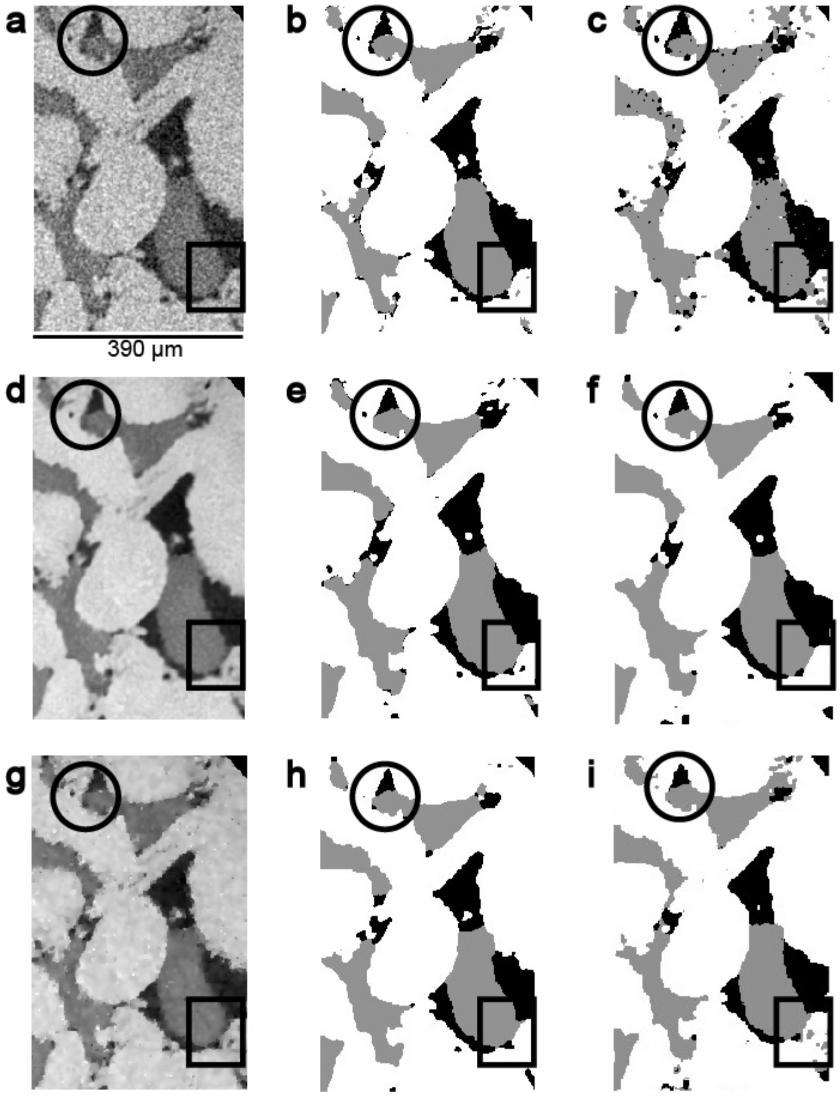

Figure S9. Magnified view of the six combinations of noise filter and segmentation. a. A raw image of sample 2 with oil, brine and rock in black, dark gray and light gray respectively. b. Segmented raw image using Trainable WEKA Segmentation. c. Segmented raw image by three-phase watershed segmentation. d. Filtered image by applying the non-local means filter. e. Segmented image using two-phase watershed segmentation after applying the non-local means filter. f. Segmented image using three-phase watershed segmentation after applying the non-local means filter. g. The image after applying an anisotropic diffusion filter. h. The segmented image using three-phase watershed segmentation after applying the anisotropic diffusion filter. i. Segmented image using three-phase Trainable WEKA Segmentation after applying the non-local means filter.

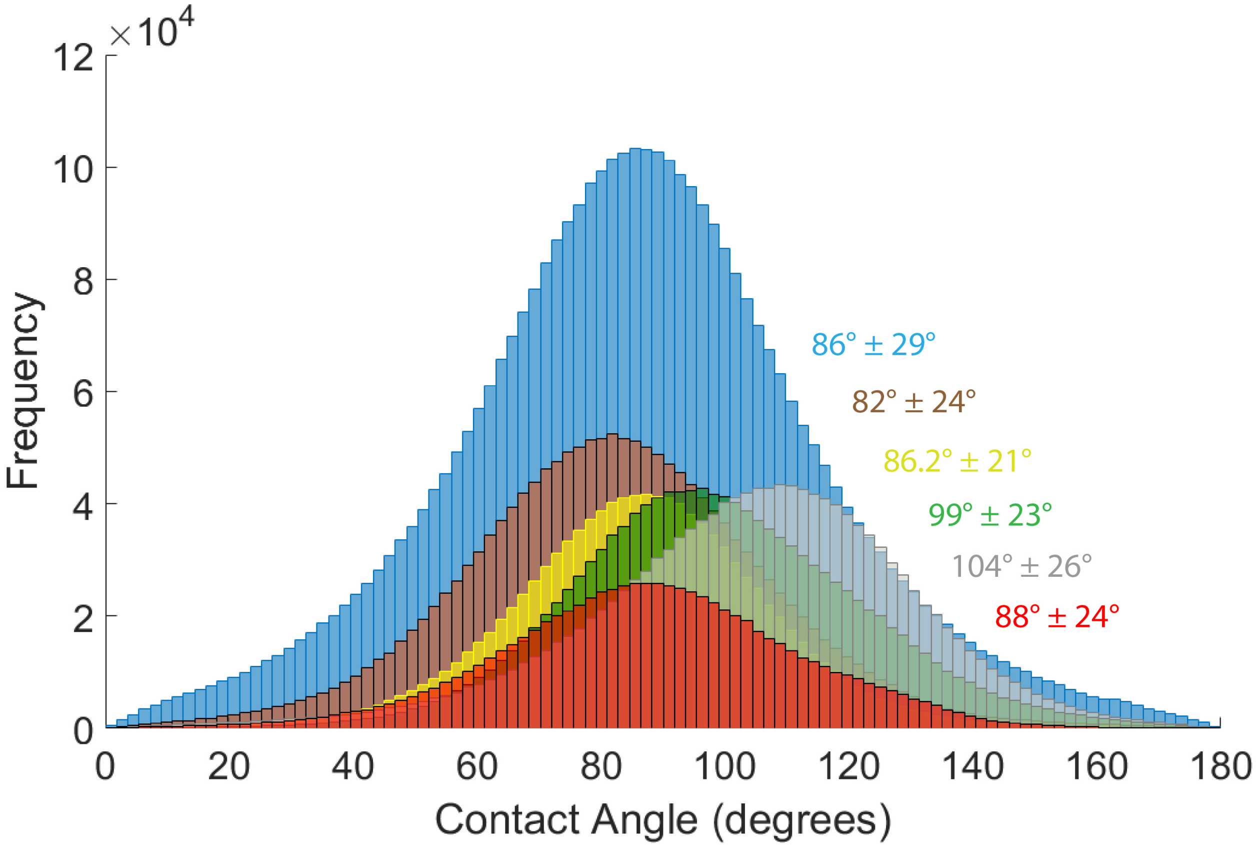

Figure S10. The measured distributions of contact angle for the six cases with mean and standard deviation. Gray is for the segmented raw image using Trainable WEKA Segmentation. Blue is for the segmented raw image using three-phase watershed segmentation. Green is for the segmented image using two-phase watershed segmentation after applying the non-local means filter. Red is for the segmented image using three-phase watershed segmentation after applying a non-local means filter. Yellow is for the segmented image using three-phase watershed segmentation after applying an anisotropic diffusion filter. Brown is for the segmented image using three-phase Trainable WEKA Segmentation after applying the non-local means filter.

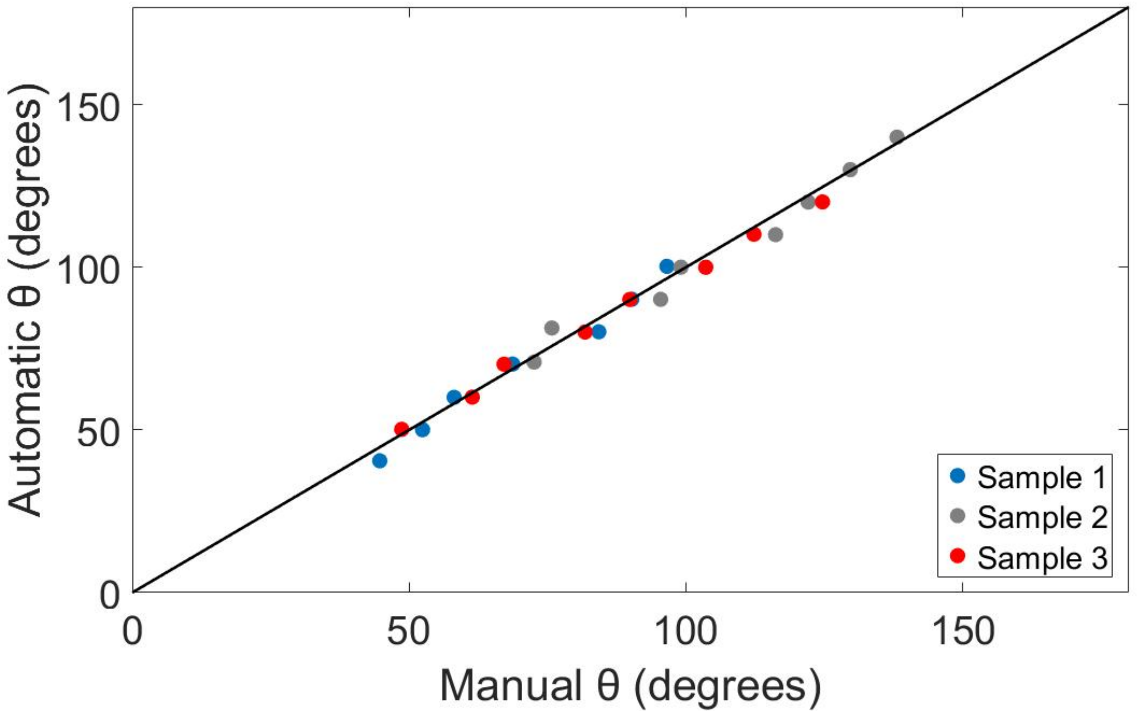

Figure S11. Comparison of manual and automatic estimates of contact angle at the same location for the three samples. Blue, gray and red are for samples 1, 2 and 3 respectively.

References

1. Andrew, M., Bijeljic, B. & Blunt, M. J. Pore-scale imaging of trapped supercritical carbon dioxide in sandstones and carbonates. *Int. J. Greenh. Gas Control.* **22**, 1–14 (2014).
2. Singh, K., Bijeljic, B. & Blunt, M. J. Imaging of oil layers, curvature and contact angle in a mixed-wet and a water-wet carbonate rock. *Water Resour. Res.* **52**, 1716–1728 (2016).
3. Blunt, M. J. Multiphase flow in permeable media: A pore-scale perspective. In *Cambridge University Press*, 188–216 (2017).
4. Plancher, H., Dorrence, S. M. & Petersen, J. C. Identification of chemical types in asphalts strongly absorbed at the asphalt–aggregate interface and their relative displacement by water. *Assoc. Asph. Paving Technol. Proc.* **46**, 151–175 (1977).
5. Buckley, J. S., Liu, Y. & Monsterleet, S. Mechanisms of wetting alteration by crude oils. *Soc. Petroleum Eng. J.* **3**, 54–61 (1998).
6. Crocker, M. E. & Marchin, L. M. Wettability and adsorption characteristics of crude-oil asphaltene and polar fractions. *J. Petroleum Technol.* **40**, 470–474 (1988).
7. Sayyouh, M. H., Hemeida, A. M., Al-Blehed, M. S. & Desouky, S. M. Role of polar compounds in crude oils on rock wettability. *J. Petroleum Sci. Eng.* **6**, 225–233 (1991).
8. Salathiel, R. A. Oil recovery by surface film drainage in mixed-wettability rocks. *J. Petroleum Technol.* **25**, 1216–1224 (1973).
9. Buckley, J. S., Liu, Y., Xie, X. & Morrow, N. R. Asphaltenes and crude oil wetting-the effect of oil composition. *Soc. Petroleum Eng. J.* **2**, 107–119 (1997).
10. Al-Menhali, A. S. & Krevor, S. Capillary trapping of CO_2 in oil reservoirs: Observations in a mixed-wet carbonate rock. *Environ. Sci. & Technol.* **50**, 2727–2734 (2016).
11. Wang, J. & Buckley, J. S. Asphaltene stability in crude oil and aromatic solvents-the influence of oil composition. *Energy & Fuels* **17**, 1445–1451 (2003).

12. Wang, J. X. & Buckley, J. S. A two-component solubility model of the onset of asphaltene flocculation in crude oils. *Energy & Fuels* **15**, 1004–1012 (2001).

13. Fernø, M. A., Torsvik, M., Haugland, S. & Graue, A. Dynamic laboratory wettability alteration. *Energy & Fuels* **24**, 3950–3958 (2010).

14. Idowu, N. *et al.* Wettability analysis using micro-CT, FESEM and QEMSCAN and its applications to digital rock physics. *Proc. Int. Symp. Soc. Core Analysts* (2015).

15. Buades, A., Coll, B. & Morel, J. M. A non-local algorithm for image denoising. *IEEE Comput. Soc. Conf. on Comput. Vis. Pattern Recognit.* **https://doi.org/10.1109/CVPR.2005.38** (2005).

16. Buades, A., Coll, B. & Morel, J. M. Nonlocal image and movie denoising. *Int. J. Comput. Vis.* **76**, 123–139 (2008).

17. Arganda-Carreras, I. *et al.* Trainable WEKA segmentation: a machine learning tool for microscopy pixel classification. *Bioinforma. (Oxford Univ Press. https://doi.org/10.1093/bioinformatics/btx180* **33**, 2424–2426 (2017).

18. Kaynig, V., Fuchs, T. & Buhmann, J. M. Neuron geometry extraction by perceptual grouping in ssTEM images. *IEEE Conf. on Comput. Vis. Pattern Recognit.* **https://doi.org/10.1109/CVPR.2010.5540029** (2010).

19. Rudyanto, R. D. *et al.* Comparing algorithms for automated vessel segmentation in computed tomography scans of the lung: the VESSEL12 study. *Med. Image Analysis* **18**, 1217–1232 (2014).

20. Wollatz, L., Johnston, S. J., Lackie, P. M. & Cox, S. J. 3D histopathology—a lung tissue segmentation workflow for microfocus X-ray-computed tomography scans. *J. Digit. Imaging* **http://doi.org/10.1007/s10278-017-9966-5** (2017).

21. Wang, Y., Lin, C. L. & Miller, J. D. Improved 3D image segmentation for X-ray tomographic analysis of packed particle beds. *Miner. Eng.* **83**, 185–191 (2015).

22. Jones, A. C. *et al.* Assessment of bone ingrowth into porous biomaterials using micro-ct. *Biomater.* **28**, 2491–2504 (2007).

23. Drelich, J., Miller, J. D. & Good, R. J. The effect of drop (bubble) size on advancing and receding contact angles for heterogeneous and rough solid surfaces as observed with sessile-drop and captive-bubble techniques. *J. Colloid Interface Sci.* **179**, 37–50 (1996).
